# A common polymorphism of *COMT* was associated with symptomatic lumbar disc herniation based on a large sample with Chinese Han ancestry

**DOI:** 10.1038/s41598-018-31240-9

**Published:** 2018-08-29

**Authors:** Hongliang Liu, Hongmou Zhao, Zhong Li, Hanzhong Xue, Jun Lu, Wei Ma

**Affiliations:** 1 Department of Orthopedics, the First Affiliated Hospital of Xi’an Jiao Tong University, Xi’an, Shaanxi China; 2 Department of Orthopedics, Honghui Hospital, Xi’an Jiaotong University, Xi’an, Shaanxi China; 3 Department of Foot and Ankle Surgery, Honghui Hospital, Xi’an Jiaotong University, Xi’an, Shaanxi China; 4 Department of Trauma Orthopedics, Honghui Hospital, Xi’an Jiaotong University, Xi’an, Shaanxi China; 5 Department of Internal Medicine, Honghui Hospital, Xi’an Jiaotong University, Xi’an, Shaanxi China

## Abstract

Lumbar disc herniation (LDH) is a common spine disease characterized by a tear in the disc ring and bulges out at the soft portion. *COMT* is a protein coding gene located at 22q11.21, and its gene product is a major mammalian enzyme involved in the degradation of catecholamines. A total of 2,678 study subjects with Chinese Han ancestry were recruited and 15 SNPs were selected for genotyping in our study subjects. A synonymous coding SNP, rs4633, was identified to be significantly associated with the disease status of LDH after adjusting for BMI (OR = 0.76, *P* = 4.83 × 10^−5^). This SNP was also identified to be significantly associated with *COMT* gene expression in three types of human tissues. Minor alleles of rs4633 (T) increased the expression of *COMT* in these 3 tissues. We have identified a significant SNP of *COMT*, rs4633, which is associated with symptomatic LDH in a large Chinese Han-based sample of the study subjects. This significant finding is further replicated by haplotypic analysis. Evidence from bioinformatics analyses have shown that rs4633 is also significantly associated with the gene expression of *COMT*. Our findings provide additional supportive evidence for an important role of *COMT* gene in the symptomatic LDH susceptibility.

## Introduction

Lumbar disc herniation (LDH) is a common spine disease characterized by a tear in the disc ring and bulging out of its soft portion^1^. The most common symptom of symptomatic LDH is sciatica, and approximately 90% of the time, sciatica is caused by symptomatic LDH^1^. Symptomatic LDH patients might also experience a numb feeling throughout one or both of their legs due to the compression of the femoral nerve root by the hernia. Symptomatic LDH and its related low back pain can be devastating for the quality of life for LDH patients. Compared to those without symptomatic LDH, patients with symptomatic LDH have heavy health care loads and incur great work loss. As a complex disease with multiple contributing factors, previous studies have identified various factors, such as age, smoking, and obesity, to be significantly associated with LDH. In addition to these environmental factors, genetics have also been proven to play an important role in the pathology of LDH. A study conducted by Sambrook *et al*. showed that genetic variations could explain approximately 50–70% of the phenotypic variations of LDH^2^. Genes including *COL11A1*^3^, *THBS2*^4^, *ASPN*^5^, *CILP*^6,7^, *COL9A3*^8^ and *MMP9*^4^ have been confirmed to be loci of susceptibility to LDH or disc degeneration. The effect size of the significant polymorphisms identified from these loci were tiny compared to the heritability of LDH, and this indicated that more genes that contributed to the risk of LDH were still not discovered and more studies are still needed in the future to unravel the genetic etiology of LDH. *COMT* is a protein coding gene comprised of 10 exons located at 22q11.21. This gene was well known for its connections with psychiatric disorders (especially schizophrenia)^9–11^. Some recent studies have shown that this gene also contributed to bone related disorders. A 2014 study conducted by Gruber *et al*. has shown that SNP rs4633 of *COMT* was significantly associated with disc degeneration in a study sample of Europeans^12^. However, to the best of our knowledge, this finding has not been replicated in any other population and therefore studies based on populations other than Europeans are necessary to validate the significant hits identified by Gruber *et al*. In this study, we aimed to investigate the genetic association between the polymorphisms of *COMT* and the symptomatic LDH in a study sample comprised of 2,678 subjects with Chinese Han ancestry. The biological functions of significant SNPs were also examined by relevant bioinformatics tools. Our findings will validate the results of previous studies based on European populations and offer insights for unraveling the underlying mechanisms of *COMT* and its association with LDH.

## Methods

### Study Subjects

In our study, 875 patients with symptomatic LDH and 1,803 normal controls were recruited from Honghui Hospital of Xi’an Jiaotong University. The patients were diagnosed by positive magnetic resonance imaging (MRI) findings for symptomatic LDH with a history of unilateral pain radiating from the back along the femoral or sciatic nerve to the corresponding dermatome of the nerve root for more than 4 weeks at least. The exclusion criteria for the patients were as follows: (1) patients with mental illness or severe dysfunction of important organs (such as heart, lung, liver, or kidney); (2) patients with blood diseases, diabetes, autoimmune diseases, or tumors; and (3) patients with a body mass index (BMI) ≤ 18.5 kg/m^2^ or with a BMI ≥ 28 kg/m^2^. The controls with good health as confirmed by physical examination were enrolled, and they had a lifetime lack of symptoms indicating symptomatic LDH, such as lumbocrural pain. Subjects with osteoarthritis, rheumatism, connective tissue diseases, previous fractures of the spine, lumbar spinal stenosis, malignancies involving the spine and poliomyelitis were excluded from the study. Lumbar sagittal MRI was conducted by using a 1.5 Tesla Magnetom unit (Siemmens AG, Germany). A sagittal T2-weighted image with a slice thickness of 5 mm, a repetition of 2500 ms, and an echo time of 90 ms was applied for all subjects. The MRI diagnosis of LDH was based on the disk extended beyond the margins of adjacent vertebral bodies, which was confirmed by two observers at least. The disc degeneration was graded by using the Schneiderman classification (based on the signal intensity): grade 1, normal signal intensity; grade 2, slightly decreased signal intensity; grade 3, diffuse signal loss; grade 4, lack of signal. The clinical data and characteristics of all the subjects were measured or recorded (Table 1). We have collected informed consent with signature for all of our study subjects. All procedures were conducted in accordance with the ethical standards of the Helsinki Declaration of 1975, revised in 2008, and the study was reviewed and approved by the Medical Ethics Committee of Honghui Hospital of Xi’an Jiaotong University.

**Table 1:** The clinical characteristics of the subjects.

Characteristics	Subjects (N = 2,678)		*P*-value
	Cases (N = 875)	Controls (N = 1,803)	
Age (years), mean ± SD	44.7 ± 10.2	44.8 ± 9.9	0.84
Gender (male/female)	604/271	1248/555	0.96
BMI (kg/m^2^), mean ± SD	24.1 ± 0.83	24.0 ± 0.84	2.9 × 10^−6^
Family history (yes/no)	99/776	144/1659	0.006
Smoking history (yes/no)	201/674	409/1394	0.91
History of alcohol intake (yes/no)	125/750	255/1548	0.97
**Grade of disc degeneration**			
2	248	NA	NA
3	463	NA	NA
4	164	NA	NA

SD: standard deviation; BMI, body mass index; NA, not available. χ^2^ tests were performed for gender, family history, smoking history and history of alcohol intake, while t tests were conducted for age and BMI.

### SNP Selection and Genotyping

We searched for all the SNPs with minor allele frequencies (MAF) ≥ 0.05 within the region of the *COMT* gene in the 1000 Genomes Project Chinese Han Beijing population database. Then, MAF ≥ 0.05 with pair-wise tagging and r^2^ ≥ 0.6 were used as the cut-off criteria during tag SNP selection, which generated 14 tag SNPs for our study. In addition, we also included SNP rs4680 as our candidate SNPs based on previous publications. Basic information on the 15 selected SNPs is summarized in Table 2. We have extracted the genomic DNA using the peripheral blood of our study subjects. The DNA extractions were conducted using Tiangen DNA extraction kit based on the protocol recommended by the manufacturer. Sequenom MassARRAY platform was utilized for SNP genotyping. The detected signals from MassARRAY platform were analyzed by software Typer 4.0 which was recommended by Sequenom^13^. About 5% of the sample was repeated for genotyping to estimate the quality of genotyping experiments. With a concordance rate of 100%, the quality of our genotyping data were guaranteed. Case control status of our study sample were blinded to the technicians during genotyping process.

**Table 2:** Results of single marker based association analyses.

CHR	SNP	BP	FUNC	MAF	HWE	A1	OR	T	*P*
22	rs3788319	19942028	intron	0.41	0.62	G	1.01	0.13	0.90
22	rs8185002	19945525	intron	0.28	0.77	G	1.01	0.21	0.84
22	rs174673	19946255	intron	0.32	0.79	C	0.96	−0.58	0.56
22	rs9332331	19947460	intron	0.08	0.28	A	0.89	−1.05	0.29
22	rs9332334	19947625	intron	0.31	0.78	C	0.99	−0.15	0.88
22	rs76157168	19951186	intron	0.19	0.94	T	1.02	0.21	0.83
22	rs112080665	19951236	intron	0.11	0.64	G	1.05	0.51	0.61
22	rs5993887	19953138	intron	0.42	0.92	T	0.97	−0.53	0.60
22	rs165767	19957037	intron	0.34	0.83	A	1.02	0.25	0.80
22	rs11912354	19959824	intron	0.47	0.85	C	1.01	0.21	0.84
**22**	**rs4633**	**19962712**	**coding-synon**	**0.27**	**0.86**	**T**	**0.76**	**−4.06**	**4.83 × 10** ^**−5**^
22	rs4680	19963748	coding-nonsy	0.36	0.50	A	1.19	2.90	0.0037
22	rs4646315	19964374	intron	0.12	1.00	C	1.04	0.41	0.68
22	rs165737	19964978	intron	0.20	0.88	T	0.99	−0.19	0.85
22	rs165728	19969500	intron	0.35	0.76	C	0.99	−0.24	0.81

HWE: *P* values of Hardy-Weinberg Equilibrium tests.; A1: Tested allele. Significant SNP was shown in bold.

### Statistical Analysis

Single marker based genetic association analyses were performed for each SNP using logistic models. According to the results from Table 1, we have included BMI as covariates in the logistic models. Bonferroni corrections were applied to address multiple comparison (*P*_threshold_ = 0.05/15 ≈ 0.0033). Plink was utilized for single marker based analysis. Q-Q plot was made to examine the potential inflation for the significance of the *P* values caused by population stratifications. In addition to single marker based analyses, we also constructed LD blocks using our data and performed haplotypic analyses. Haploview was utilized for these analyses. In addition to the genetic association analyses, we have also conducted an association analysis for symptomatic LDH cases only to investigate the potential link between targeted SNPs and the disc degeneration.

### Bioinformatics Analysis

To further examine the potential functional significance of those significant hits in genetic association analysis, we performed bioinformatics analyses through online databases. Significant SNPs identified in genetic association analyses were checked in RegulomeDB (http://regulomedb.org/). RegulomeDB is an online tool to predict the potential functional significance of SNPs by integrating Encode data. It returns a score, which ranges from 1 to 6, to label the regulatory function of the SNPs regarding the gene expression and translation. In addition, we have also investigated the potential effects of targeted SNPs on *COMT* gene expression in multiple human tissues using the GTEx database (https://www.gtexportal.org/home/).

## Results

### Significant hits in genetic association analyses

The clinical characteristics of the study subjects are summarized in Table 1. Significant differences in body mass index (BMI) can be identified for symptomatic LDH patients and controls. All the selected 15 SNPs were in Hardy-Weinberg equilibrium in our control sample. A synonymous coding SNP, rs4633, was identified to be significantly associated with the disease status of symptomatic LDH after adjusting for BMI (OR = 0.76, *P* = 4.83 × 10^−5^, Table 2). No signs of inflation caused by population stratification can be identified from the Q-Q plot for the results of the single marker-based association analyses (Supplemental Fig. S1).

A total of 4 linkage disequilibrium (LD) blocks were constructed based on our data (Supplemental Fig. S2). Haplotypic association analyses were performed for these LD blocks and have validated the results of the single marker based analyses. A 3-SNP haplotype, which included the SNP rs4633, was identified to be significantly associated with symptomatic LDH (rs4633|rs4680|rs4646315, *P* = 2.23 × 10^−28^, Table 3). No significant association can be identified between rs4633 and the disc degeneration using the data of symptomatic LDH patients only (χ^2^ = 5.99, *P* = 0.1999, Supplemental Table S1).

**Table 3:** Results of haplotype based analysis.

LOCUS	HAPLOTYPE	F_A	F_U	CHISQ	DF	*P*	SNPs
H1	OMNIBUS	—	—	0.19	2	0.91	rs3788319|rs8185002
H1	GG	0.28	0.27	0.06	1	0.81	rs3788319|rs8185002
H1	GT	0.14	0.13	0.09	1	0.76	rs3788319|rs8185002
H1	AT	0.59	0.59	0.18	1	0.67	rs3788319|rs8185002
H2	OMNIBUS	—	—	1.03	2	0.60	rs5993887|rs165767
H2	TA	0.34	0.33	0.03	1	0.86	rs5993887|rs165767
H2	TG	0.08	0.08	1.03	1	0.31	rs5993887|rs165767
H2	CG	0.59	0.58	0.16	1	0.69	rs5993887|rs165767
**H3**	**OMNIBUS**	**—**	**—**	**131.8**	**3**	**2.23 × 10** ^**−28**^	**rs4633|rs4680|rs4646315**
**H3**	**TAC**	**0.12**	**0.12**	**0.00**	**1**	**0.96**	**rs4633|rs4680|rs4646315**
**H3**	**TAG**	**0.11**	**0.16**	**28.45**	**1**	**9.62 × 10** ^**−8**^	**rs4633|rs4680|rs4646315**
**H3**	**CAG**	**0.16**	**0.07**	**116.21**	**1**	**4.36 × 10** ^**−27**^	**rs4633|rs4680|rs4646315**
**H3**	**CGG**	**0.61**	**0.65**	**7.86**	**1**	**0.005**	**rs4633|rs4680|rs4646315**
H4	OMNIBUS	—	—	0.20	2	0.91	rs165737|rs165728
H4	TC	0.19	0.20	0.20	1	0.66	rs165737|rs165728
H4	CC	0.16	0.16	0.02	1	0.89	rs165737|rs165728
H4	CT	0.65	0.64	0.07	1	0.79	rs165737|rs165728

Significant results were highlighted in bold. F_A: frequency in cases; F_U: frequency in controls; DF: degree of freedom.

### RegulomeDB score and eQTL results from GTEx

The regulomeDB score for rs4633 is 2b, which indicates that this SNP has a relatively significant regulatory function on gene expression and translation of *COMT*. According to the records of regulomeDB, this SNP located at a DNase I hypersensitivity region based on the data of 125 cell types from ENCODE. In addition, the location of this SNP belongs to a transcription factor binding motif and is an important protein binding site for multiple human proteins.

**Figure 1:**
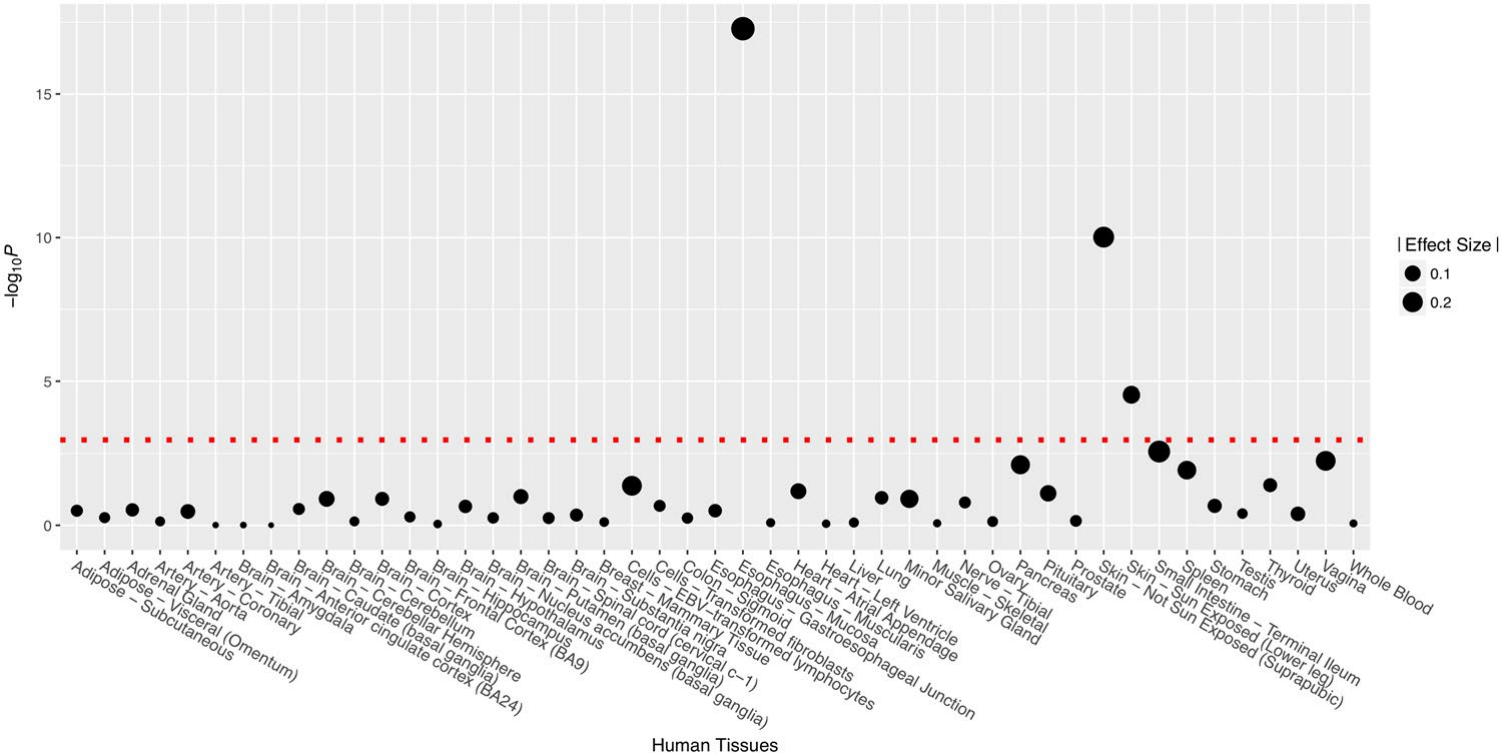
eQTL data of *COMT* for rs4633 in multiple human tissues. Data were extracted from GTEx database. *P* value threshold obtained through bonferroni correction was indicated by red dotted line.

The eQTL data of rs4633 in 47 human tissues were extracted from the GTEx database. The SNP rs4633 was identified to be significant (*P*_threshold_ = 0.05/47 ≈ 0.001) in three types of human tissues (Fig. 1): the mucosa of the esophagus, the skin of lower leg (sun exposed) and the skin of suprapubic (non-exposed for sun). Minor alleles of rs4633 (T) increased the expression of *COMT* in all 3 tissues (Supplemental Fig. S3).

## Discussion

Catechol-O-methyltransferase, which is encoded by *COMT*, is a major mammalian enzyme involved in the degradation of catecholamines. It assists the process of transferring a methyl group from S-adenosyl-methionine to a hydroxyl group on a catechol nucleus^14^. In recent years, candidate gene studies have successfully identified many susceptible loci for complex diseases^15–21^. Because of its molecular biological function, which is related to multiple neurotransmitters, a significant number of previous studies have tried to identify its susceptibility to brain related disorders, such as schizophrenia^9–11^, anorexia nervosa^22^ and mental retardation^23^. Unlike these brain-related disorders, very limited studies have tried to establish the link between *COMT* and human bone-related disorders. In a 2007 study conducted by Stolk *et al*., SNP rs4680 of *COMT* was identified to be significantly associated with osteoporotic and fragility fracture risk in European men after adjusting for age, height, weight, and bone mineral density (BMD)^24^. Two other studies have reported significant associations between polymorphisms of *COMT* and the treatment outcome of lumbar degenerative disc disease and low back pain^25–27^. SNP rs4633, the same SNP identified to be significant in our study, has been reported to be related to disc degeneration in a 2014 study conducted by Gruber *et al*.^12^. Considering that only SNPs analyses are not sufficient to reach a reliable conclusion^28–32^, we have further analyzed the haplotype, of which the results are similar with that of SNPs analyses. To the best of our knowledge, this study is the first one to establish the link between symptomatic LDH and polymorphisms of *COMT* in the Chinese population. We have successfully replicated the results reported by Gruber *et al*. using sample from different populations. Unlike the brain related disorders, which have a clear connection with the biological function of Catechol-O-methyltransferase, its connection with bone related disorders has not been clearly established. More studies focusing on pathological mechanisms of LDH and disc degeneration are needed in the future.

The significant SNP identified in our study, rs4633, is a synonymous coding variant. Our bioinformatics analysis using regulomeDB has shown that it might have significant regulatory function on the gene expression of *COMT*. Further eQTL data from GTEx also provides some evidence on its role in the expression of *COMT*. Nevertheless, it is still too early to conclude that rs4633 is a genetic polymorphism that contributes directly to the risk of LDH (but not a surrogate of some underlying variant(s)). An interesting observation is that most of the previous association studies targeting *COMT* (including both brain-related disorders and bone-related disorders) have reported rs4680 (also referred as Val-158-Met), but not rs4633, to be a significant hit. Unlike rs4633, rs4680 is a non-synonymous coding variant, which will cause a change in the amino acid gene product of *COMT*. However, in the study conducted by Gruber *et al*., although both rs4680 and rs4633 were genotyped, only rs4633 was found to be significantly associated with disc degeneration. The explanation for this discordance is unknown. In our study, only rs4633 was identified to be significantly associated with LDH, which supported findings from the study of Gruber *et al*.

One important thing to note is that the *P* values of single SNP associations in Table 2 were much weaker than the *P* values of haplotype analysis in Table 3. The results suggested that the association signal may be contributed by haplotypes rather than single SNP. If we only considered the two functional SNPs, rs4633 and rs4680, in this haplotype, we could find that the global association was driven by haplotype CA. There is a possible explanation for that in potential molecular effects of these SNPs. Furthermore, we could hypothesize that both A allele of rs4680 and C allele of rs4633 are related to the low protein abundance of COMT and in turn will affect the activity of COMT, which have been reported in a previous study^33^. Give of the lack of direct supportive biological evidence in our study, the hypothesis would be desired to verify in the future study.

A potential limitation for our study is that the SNPs selected for genotyping might not provide enough information on the coverage of *COMT*. There are approximately 2,000 variants located within the *COMT* gene regions, and even if we considered the LD pattern of the SNPs, 15 SNPs are not enough to represent a very limited number of SNPs. In addition, all our selected SNPs were located within the gene region of *COMT*. However, the evidence has shown that the genomic regions located several kbs up/down-stream of the targeted gene might have important regulatory effects. Failing to consider variants of these regions might miss important genetic signals for LDH susceptibility. Therefore, additional studies with more SNPs and rare/low frequency variants based on targeted sequencing are needed to conclude that *COMT* is involved in pathological process of LDH. Another potential limitation is that our selection strategy for study subjects might introduce bias for this study. The prevalence of LDH in the asymptomatic population is high, but asymptomatic LDH patients hardly go to the hospital for diagnosis and treatment, which also makes it more difficult for us to recruit the asymptomatic LDH patients. Therefore, in this study, we only focus on symptomatic LDH patients. It is also partly uncertain whether our results are applicable to the general population. Our data are of the interest harboring further etiological and genetic study of LDH. However, given that the development of LDH is an extremely complex pathophysiology process involving many genes and factors, further studies are necessary to confirm our results.

In summary, in this study, we have identified a significant SNP of *COMT*, rs4633, that is associated with the disease status of symptomatic LDH in a large Han Chinese-based sample of study subjects. This significant finding is further replicated by haplotypic analysis. Evidence from the bioinformatic analyses have shown that rs4633 is also significantly associated with the gene expression of *COMT*. More studies focusing on the pathological mechanisms of LDH and *COMT* are still needed in the future to unravel the role that *COMT* and its gene products played in the onset and development of LDH.

## Electronic supplementary material

Supplementary materials

## References

[1] Ernst CW, Stadnik TW, Peeters E, Breucq C, Osteaux MJC (2005). Prevalence of annular tears and disc herniations on MR images of the cervical spine in symptom free volunteers. European journal of radiology.

[2] Sambrook PN, MacGregor AJ, Spector TD (1999). Genetic influences on cervical and lumbar disc degeneration - A magnetic resonance imaging study in twins. Arthritis and rheumatism.

[3] Mio F (2007). A functional polymorphism in COL11A1, which encodes the alpha 1 chain of type XI collagen, is associated with susceptibility to lumbar disc herniation. American journal of human genetics.

[4] Hirose Y (2008). A functional polymorphism in THBS2 that affects alternative splicing and MMP binding is associated with lumbar-disc herniation. American journal of human genetics.

[5] Song YQ (2008). Association of the asporin D14 allele with lumbar-disc degeneration in Asians. American journal of human genetics.

[6] Seki S (2005). A functional SNP in CILP, encoding cartilage intermediate layer protein, is associated with susceptibility to lumbar disc disease. Nature genetics.

[7] Virtanen M (2007). Phenotypic and population differences in the association between CILP and lumbar disc disease. Journal of medical genetics.

[8] Paassilta P (2001). Identification of a novel common genetic risk factor for lumbar disk disease. Jama-J Am Med Assoc.

[9] Shifman S (2002). A highly significant association between a COMT haplotype and schizophrenia. American journal of human genetics.

[10] Bray NJ (2003). A haplotype implicated in schizophrenia susceptibility is associated with reduced COMT expression in human brain. American journal of human genetics.

[11] Palmatier MA (2004). COMT haplotypes suggest P2 promoter region relevance for schizophrenia. Mol Psychiatr.

[12] Gruber HE (2014). A Novel Catechol-O-Methyltransferase Variant Associated with Human Disc Degeneration. International journal of medical sciences.

[13] Guan F (2012). Association study of a new schizophrenia susceptibility locus of 10q24.32-33 in a Han Chinese population. Schizophr Res..

[14] Chen JS (2004). Functional analysis of genetic variation in catechol-o-methyltransferase (COMT): Effects on mRNA, protein, and enzyme activity in postmortem human brain. American journal of human genetics.

[15] Guan F (2012). Association of PDE4B polymorphisms and schizophrenia in Northwestern Han Chinese. Hum Genet..

[16] Guan F (2014). MIR137 gene and target gene CACNA1C of miR-137 contribute to schizophrenia susceptibility in Han Chinese. Schizophr Res..

[17] Chen G, Guan F, Lin H, Li L, Fu D (2015). Genetic analysis of common variants in the HDAC2 gene with schizophrenia susceptibility in Han Chinese. Journal of human genetics..

[18] Guan F (2015). Evaluation of genetic susceptibility of common variants in CACNA1D with schizophrenia in Han Chinese. Scientific reports..

[19] Zhang B (2015). Common variants in SLC1A2 and schizophrenia: Association and cognitive function in patients with schizophrenia and healthy individuals. Schizophr Res..

[20] Guan F (2016). Evaluation of association of common variants in HTR1A and HTR5A with schizophrenia and executive function. Scientific reports..

[21] Guan F (2016). Evaluation of voltage-dependent calcium channel γ gene families identified several novel potential susceptible genes to schizophrenia. Scientific reports..

[22] Frisch A (2001). Association of anorexia nervosa with the high activity allele of the COMT gene: a family-based study in Israeli patients. Mol Psychiatr.

[23] Zhang K (2007). An association study between cathechol-O-methyltransferase gene and mental retardation in the Chinese Han population. Neuroscience Letters.

[24] Stolk L (2007). The catechol-O-methyltransferase Met158 low-activity allele and association with nonvertebral fracture risk in elderly men. The Journal of clinical endocrinology and metabolism.

[25] Omair A, Lie BA, Reikeras O, Holden M, Brox JI (2012). Genetic contribution of catechol- O-methyltransferase variants in treatment outcome of low back pain: a prospective genetic association study. BMC Musculoskelet Disord.

[26] Dai F (2010). Association of catechol-O-methyltransferase genetic variants with outcome in patients undergoing surgical treatment for lumbar degenerative disc disease. The spine journal: official journal of the North American Spine Society.

[27] Omairs A (2015). Catechol-O-methyltransferase (COMT) gene polymorphisms are associated with baseline disability but not long-term treatment outcome in patients with chronic low back pain. Eur Spine J..

[28] Guan F (2013). A population-based association study of 2q32.3 and 8q21.3 loci with schizophrenia in Han Chinese. Journal of psychiatric research..

[29] Yang H (2013). 4q22.1 contributes to bone mineral density and osteoporosis susceptibility in postmenopausal women of Chinese Han population. PloS one..

[30] Guan F (2016). Two-stage association study to identify the genetic susceptibility of a novel common variant of rs2075290 in ZPR1 to type 2diabetes. Scientific reports..

[31] Guan F (2016). Two-stage replication of previous genome-wide association studies of AS3MT- CNNM2-NT5C2 gene cluster region in a large schizophrenia case-control sample from Han Chinese population. Schizophr Res..

[32] Jia X (2016). Two-stage additional evidence support association of common variants in the HDAC3 with the increasing risk of schizophrenia susceptibility. American journal of medical genetics. Part B, Neuropsychiatric genetics..

[33] Tsao D (2011). Disruptive mRNA folding increases translational efficiency of catechol-O-methyltransferase variant. Nucleic Acids Res..

