# Supplementary material for: A common polymorphism of COMT was associated with symptomatic lumbar disc herniation based on a large sample with Chinese Han ancestry
Source: Sci Rep. 2018 Aug 29;8:13000. doi: 10.1038/s41598-018-31240-9 (PMC6115408; doi:10.1038/s41598-018-31240-9)
Supplement: Supplementary file 1 — Supplementary materials [file 41598_2018_31240_MOESM1_ESM.docx]

***Title***: A common polymorphism of *COMT* was associated with symptomatic lumbar disc herniation based on a large sample with Chinese Han ancestry

***Author names and affiliations***: Hongliang Liu ^1,2^, Hongmou Zhao ^3^, Zhong Li ^4^, Hanzhong Xue ^4^, Jun Lu ^5^ and Wei Ma ^1^

^1^ Department of Orthopedics, the First Affiliated Hospital of Xi'an Jiao Tong University, Xi'an, Shaanxi, China;

^2^ Department of Orthopedics, Honghui Hospital, Xi’an Jiaotong University, Xi'an, Shaanxi, China;

^3^ Department of Foot and Ankle Surgery, Honghui Hospital, Xi’an Jiaotong University, Xi'an, Shaanxi, China;

^4^ Department of Trauma Orthopedics, Honghui Hospital, Xi’an Jiaotong University, Xi'an, Shaanxi, China;

^5^ Department of Internal Medicine, Honghui Hospital, Xi’an Jiaotong University, Xi'an, Shaanxi, China

***Corresponding Author***:

Jun Lu, Department of Internal medicine, Honghui Hospital, Xi’an Jiaotong University, No.555, Youyi East Road, Xi'an, Shaanxi, China, 710054.

Tel: 86-29-88418009; Fax: 86-29-88418009; E-mail: [junluhh@163.com](mailto:junluhh@163.com)

Wei Ma, Department of Orthopedics, the First Affiliated Hospital of Xi'an Jiao Tong University, No. 277 Yanta West Road, Xi’an, Shaanxi, China, 710061.

Tel.:+86-029-85323935; Fax: +86-029-85323935; E-mail: [mawwei60@163.com](mailto:mawwei60@163.com)


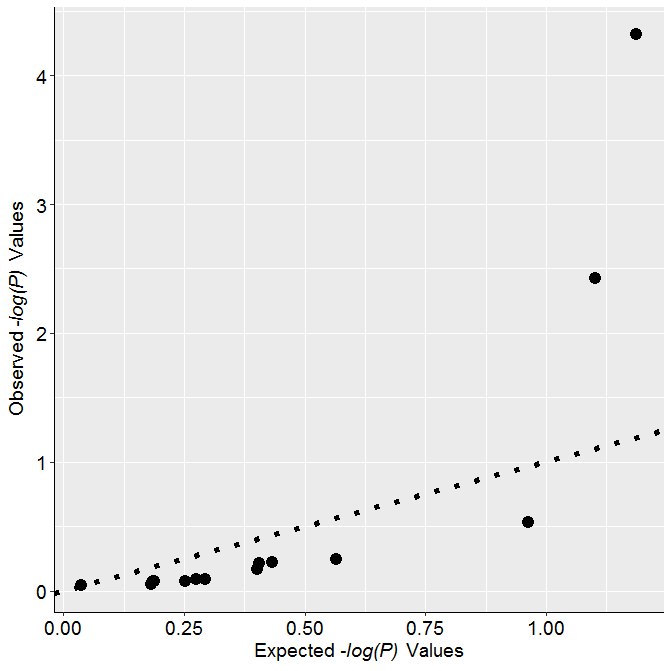
Supplemental Figure S1. Q-Q plot for the results of single marker based association analysis. No signs of inflations of association signals can be identified from this plot.


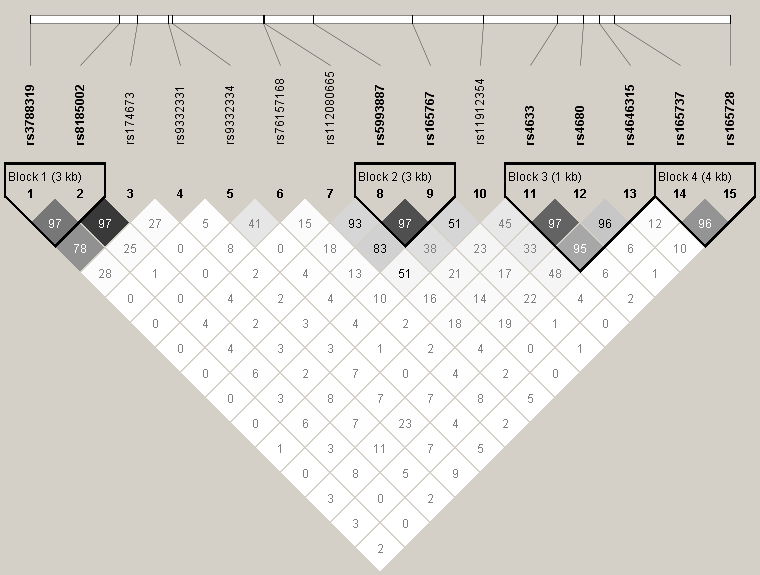


Supplemental Figure S2. LD blocks of the 15 selected SNPs in *COMT*. Values of D’ were indicated in each square and served as color scheme in this plot.


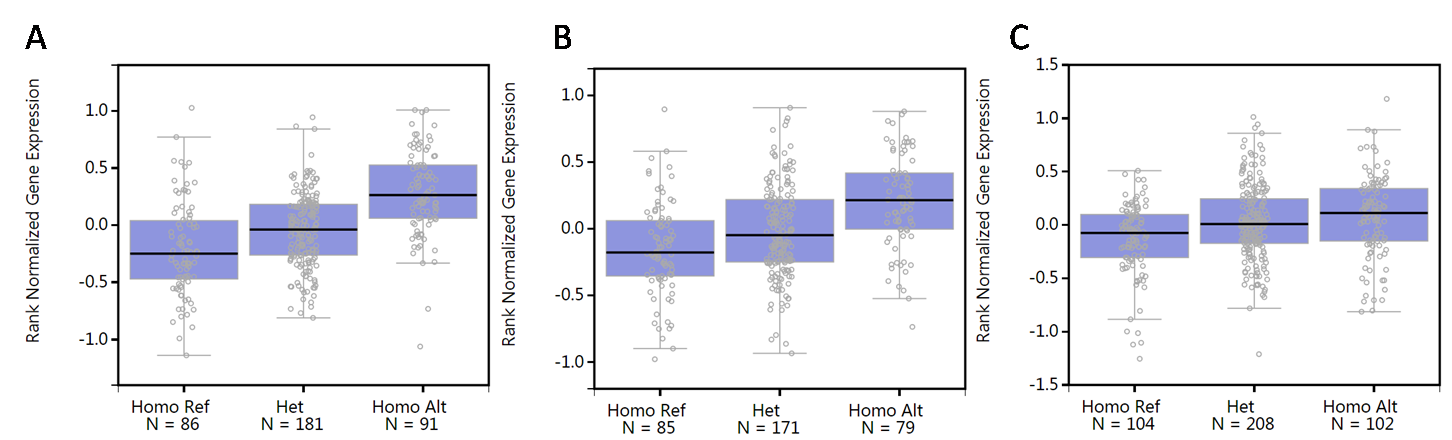


Supplemental Figure S3. Association between rs4633 and gene expressions of *COMT*. A. mucosa of esophagus; B. skin of lower leg (sun exposed). C. skin of suprapubic (non-exposed for sun).

Supplemental Table S1. Relationship between genotypes of rs4633 and the disk degeneration grades.

| Genotypes of rs4633 | Grades of disk degeneration | | | χ^2^, *P* |
| --- | --- | --- | --- | --- |
|  | 2 (N=248) | 3 (N=463) | 4 (N=164) |  |
| CC (N=517) | 149 (29%) | 278 (54%) | 90 (17%) |  |
| CT (N=315) | 87 (28%) | 158 (50%) | 70 (22%) |  |
| TT (N=43) | 12 (28%) | 27 (63%) | 4 (9%) | 5.99, 0.1999 |
